# Sensitivity of *Botrytis cinerea* Isolates Complex to Plant Extracts

**DOI:** 10.3390/molecules26154595

**Published:** 2021-07-29

**Authors:** Lina Dėnė, Alma Valiuškaitė

**Affiliations:** Laboratory of Plant Protection, Institute of Horticulture, Lithuanian Research Centre for Agriculture and Forestry, Kauno st. 30, LT-54333 Babtai, Lithuania; alma.valiuskaite@lammc.lt

**Keywords:** biocontrol, biopesticides, cinnamon extract, grey mould

## Abstract

New agricultural strategies aim to reduce the use of pesticides due to their damage to the environment and humans, and the caused resistance to pathogens. Therefore, alternative sources of antifungal compounds from plants are under investigation lately. Extracts from plants have a wide composition of chemical compounds which may complicate the development of pathogen resistance. *Botrytis cinerea*, causing grey mould, is an important horticultural and ornamental pathogen, responsible for the relevant yield and quality losses. *B. cinerea* isolated from a different plant host may differ in the sensitivity to antifungal substances from plants. Assessing the importance of research covering a wide range of pathogens for the rapid development of biopesticides, this study aims to determine the sensitivity of the *B. cinerea* isolate complex (10 strains) to plant extracts, describe morphological changes caused by the extract treatment, and detect differences between the sensitivity of different plant host isolates. The results showed the highest sensitivity of the *B. cinerea* isolates complex to cinnamon extract, and the lowest to laurel extract. In contrast, laurel extract caused the most changes of morphological attributes in the isolates. Five *B. cinerea* isolates from plant hosts of raspberry, cabbage, apple, bell pepper, and rose were grouped statistically according to their sensitivity to laurel extract. Meanwhile, the bell pepper isolate separated from the isolate complex based on its sensitivity to clove extract, and the strawberry and apple isolates based on their sensitivity to cinnamon extract.

## 1. Introduction

New strategies for reducing pesticide use are an inevitable, however, scientifically and practically demanding challenge. In the case of fungicides, it is already known that they have adverse effects on the environment and humans [1,2]. In the long run, they also caused pathogen resistance [3,4]. If not adequately managed, widespread disease control methods can become ineffective in the long run. With all the knowledge about pesticide residues in food, consumers tend to choose higher quality and more environmentally and human-safe products [5]. Therefore, alternative sources of antifungal substances are under investigation lately [6,7,8,9,10,11]. One alternative source is active compounds obtained from plants [12], such as botanical pesticides.

One of the advantages of plant-based pesticides is their biological activity from many accumulated chemical compounds, which result in a less expected pathogen resistance [13]. The sensitivity of pathogens to active compounds from plants has been described to some extent in the literature [2,7,14,15]. Morphological changes in fungal structures were observed after treatment with alternative antifungal compounds: salt solutions [16], zinc oxide nanoparticles [6], specific light spectrum [17], essential oils [1] or extracts [18], and their active components [19]. As demand for the replacement of synthetic fungicides is extremely high [14], in vitro antifungal studies should be expanded/continued to group plant materials according to their antifungal effect on the pathogen complex. This would increase the research and development of high-impact bioactive substances from plants against fungal pathogens.

*Botrytis cinerea* is known to develop fungicide resistance [4,20,21,22]. This pathogen infects many plant species and causes grey mould [23,24,25] during the growth phase and after harvest [18]. It is responsible for one of the most significant economic losses of horticultural crop production [1,4,10]. Genetic differences between *B. cinerea* cryptic species group II isolates from different plant hosts were indicated in previous studies [23]. Therefore, we would like to emphasize the importance of evaluating the sensitivity of isolates from different plant hosts or even different plant parts in the search for new active substances, as this pathogen is not host-specific, and is virulent independently from plant species [26]. Natural substances with broad antifungal properties, affecting a wide range of pathogens, harmful to various plants, should be considered for further studies as this would fasten the development of new products that could equate and supplement the fungicides used for *B. cinerea* control nowadays.

Therefore, the study of pathogens from different plant hosts should become an integral part of plant bioactive substances studies. As *B. cinerea* is harmful to various horticultural and ornamental crops [25], our study focused on isolates from several plant host species. Furthermore, we hypothesise that plant extracts cause morphological changes to pathogen colonies, which could be noticeable visually. An initial visual assessment of the pathogen is important for further, in-depth studies to determine the mechanism of action of the active substances. Our study aimed to determine the sensitivity of the *B. cinerea* isolate complex to plant extracts, evaluate the morphological changes caused by the extract treatment, and detect the differences between the sensitivity of different plant host isolates. The collected data can be further used to develop plant-based antifungal products with significant effects on *B. cinerea*.

## 2. Results

### 2.1. Sensitivity of B. cinerea Isolates Complex to Plant Extracts

This study investigated the sensitivity of the *B. cinerea* isolates complex to plant extracts under different concentrations. The isolates complex showed the highest sensitivity to cinnamon extract. All isolates showed no visual growth at an 800 µL/L concentration, 60% at 600 µL/L concentration, and 20% at 500 µL/L concentration (Table 1). *B. cinerea* isolates BC1 (strawberry) and BC6 (apple) were sensitive to the lowest concentration of cinnamon extract. After treatment with 800 µL/L of clove extract, the growth of 70% of the isolates was suppressed (radial colony growth was equal zero, Table 1). The isolate from the strawberry was the most sensitive to clove extract as the growth *B. cinerea* from this plant host was not visible from 500 µL/L concentration. Meanwhile, 30% of the isolates (plant hosts—apple, leek, and rose) still expressed growth at the highest investigated concentration of clove extract (800 µL/L). Opposite to clove and cinnamon extracts, the *B. cinerea* complex of isolates was not sensitive to laurel extract, 4200 µL/L concentration was not efficient to inhibit none of the isolates from ten plant hosts. However, *B. cinerea* from strawberry and apple showed sensitivity to laurel extract at earlier days of investigation. Overall, these two isolates had the highest sensitivity to extracts, examined in this study. Statistical differences in each isolate’s sensitivity to different concentrations of the extract are presented in Table 1.

**Figure 1:**
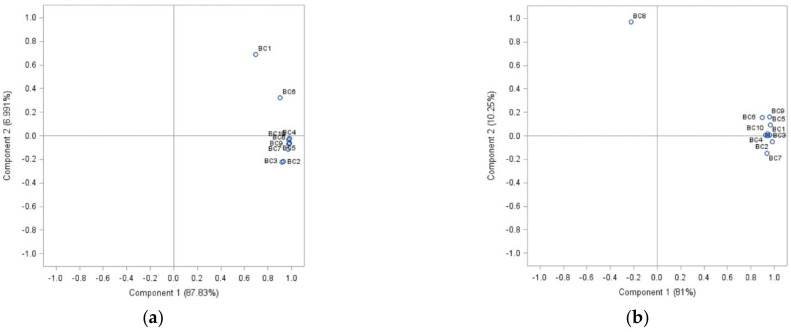

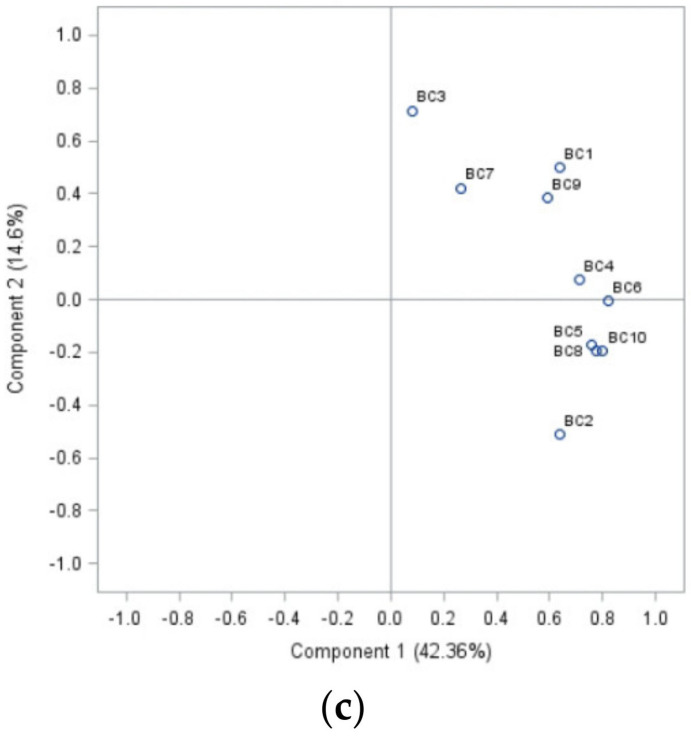
Principal components (*p* < 0.05), showing differences between the sensitivity of separate *Botrytis cinerea* isolates from ten different plant hosts complex (BC1-BC10) to cinnamon extract (**a**), clove extract (**b**), and laurel extract (**c**).

An examination of the *Botrytis cinerea* isolate complex with PCA showed a visualization of differences between the sensitivity of isolates belonging to the complex used in this study after treatment with cinnamon, clove, and laurel extracts (Figure 1).

Analysing the sensitivity of isolates to cinnamon extract, the first principal component described 87.83% of the variance (Figure 1a). Isolates BC1 (strawberry) and BC6 (apple) separated from the other isolates group according to their sensitivity to different concentrations of cinnamon extract. An analysis of treatment with clove extract, PCA1, represented 81% of the data, and PCA2 10.25% (91.25% overall) (Figure 1b). *B. cinerea* from bell pepper (BC8) differed from other isolates’ sensitivity compared to the sensitivity to clove extract. Half of the isolates formed a group based on their similarities and differences in laurel extract sensitivity. Strawberry and leek isolates (BC1 and BC9) formed a separate group. Additionally, individual differences were indicated in onion (BC2), tomato (BC3) and grape (BC7) isolates sensitivity to laurel extract. In this case, 56.96% of total data variance was explained (PCA1 and PCA2—42.36% and 14.6%, respectively) (Figure 1c).

### 2.2. Morphological Attributes of the Isolates

**Figure 2:**
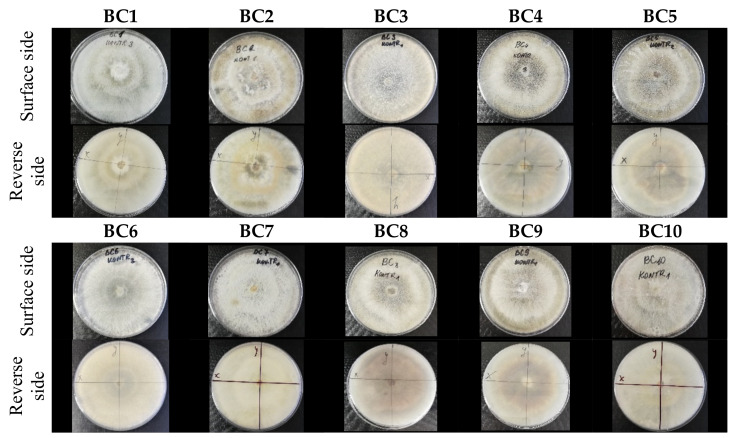
Colony morphology of *B. cinerea* control isolates grown for 7 days on PDA, at 22 °C in the dark.

*Botrytis cinerea* isolates with visible colony growth after treatment with extracts were compared to the control plates (Figure 2), where no extract was added to the medium. Observed changes of type of mycelium and colony colour are presented in Table 2. Clove and laurel extracts caused the most mycelium changes of the *B. cinerea* complex. With laurel extract, mycelium changes in isolate BC3 were observed from 3600 µL/L, and from 3200 µL/L in BC7. All investigated extracts similarly affected colour changes in isolate colonies. Colour changes were visible from treatment with 300 µL/L concentration of cinnamon extract, and from 3200 µL treatment with laurel extract. Furthermore, clove extract induced pigmentation of the medium in the most of *B. cinerea* plates, and treatment with cinnamon and laurel extracts had no significant effect.

**Figure 3:**
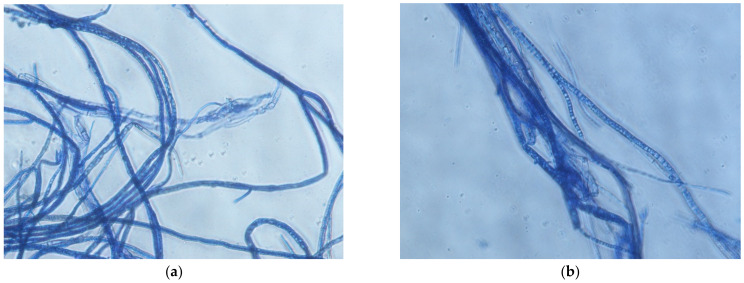
Hyphae morphology of *B. cinerea* isolate from apple, grown on PDA with 3600 µL/L (**a**) and 4200 µL/L (**b**) observed under 40× magnification.

*B. cinerea* isolates complex colonies were examined for the presence of conidia. Conidia were detected in most of the isolates, except for BC1 (strawberry) and BC6 (apple) (Table 3). The conidia length and width in control colonies of *B. cinerea* isolates varied—17.36–20.91 µm and 13.55–15.82 µm, respectively. Overall, no significant differences were observed between the control colonies conidia size and cinnamon extract-treated colonies. An amount of 400 and 600 µL/L clove extract treatment resulted in smaller conidia in examined samples of BC2 isolate (plant host—onion). Meanwhile, other isolates produced conidia in a similar size as the control. Laurel extract-treated isolates varied in conidia size the most compared to all extract treatments. Significantly smaller conidia were found in BC2, BC4 and BC8 isolates (onion, raspberry, bell pepper) colonies, and the BC7 (grape) isolate had a higher length and width of conidia compared to the control. Shrunk, twisted hyphae with coagulated inner structures were observed under the microscope after treatment with different concentrations of the examined extracts. An example of the *B. cinerea* hyphae after treatment with laurel extract is shown in Figure 3.

## 3. Discussion

In previously reported studies, the sensitivity of *B. cinerea* from various plant hosts was investigated and antifungal concentrations of extracts and essential oils were determined [1,10,11,14,15]. Mycelial growth of *B. cinerea* from tomato was inhibited by origanum essential oil at 12.8 µg/mL and lavender and rosemary essential oils at 25.6 µg/mL [1]. In another tomato *B. cinerea* study, the pathogen expressed high sensitivity to 400 and 500 mg/mL concentration of the *Vernonia amygdalina* dichloromethane extract [18]. Strawberry *B. cinerea* showed sensitivity to *Cymbopogon martinii* and *Mentha spicata* essential oils between 250 and 500 µL/L [10], and to *Aloysia citriodora*, *C. winterianus*, *Lippia alba* and *Ocimum americanum* oils at 0.8–1.4 mL/L [5].

In our study, the *B. cinerea* pathogen complex, consisting of ten strains from different plant hosts, was strongly sensitive to clove and cinnamon extract in vitro. Isolates from strawberry and apple (BC1 and BC6) significantly differed from other isolates in the complex as they expressed a sensitivity to the lowest concentration of cinnamon extract. The strawberry isolate (BC1) was also more sensitive to clove extract; however, other isolates were inhibited only with the highest tested concentration or still expressed growth. These varied sensitivity results illustrate the need for the investigation of more than one strain of the pathogen as the range of the antifungal concentration of the extract was observed individual to every plant host isolate and may be too low to inhibit all of the complex. Similarly, to our results, four *B. cinerea* isolates (plant hosts lettuce, strawberry, cucumber, and pepper) had a high sensitivity to cinnamon extract in Wahab et al.’s [8] study. The full inhibition of *B. cinerea* (kiwi fruit) growth was reached at 1000 µg/mL of laurel essential oil, extracted with supercritical carbon dioxide [14], and the growth of *Aspergillus carbonarius* was stopped with 0.3% of laurel essential oil [2]. In contrast, neither of the isolates from our *B. cinerea* complex were sensitive to laurel extract. Despite relatively high tested concentrations, it should be increased in further in vitro studies.

Isolates BC4, BC5, BC6, BC8, and BC10 (plant hosts: raspberry, cabbage, apple, bell pepper, and rose) were grouped statistically according to their growth feature after laurel extract treatment with different concentrations. Three of the isolates in the formed group had the transposa genotype, and two Vacuma. Meanwhile, only the bell pepper isolate (BC8) separated from the isolate complex after treatment with different clove extract concentrations—and strawberry (BC1) and apple (BC6) isolates—after treatment with different cinnamon extract concentrations. Our isolate complex had a higher sensitivity to cinnamon and clove extract compared to the one described in da Silva et al.’s [11] study; there, *B. cinerea* isolated from strawberries was inhibited by essential oils of *Eucalyptus staigeriana* and. *E. urograndis* using concentrations of 2000 and 4000 µL/L, respectively. Completely inhibited growth of *B. cinerea* from grapevine fruit was also observed at a lower—1500 µL/L—concentration of *Eucalyptus* sp. and *Zhumeria majdae* oils in another study [15]. Meanwhile, no sensitivity was observed at the highest (4200 µL/L) concentration of laurel extract, considering it less effective against *B. cinerea* than oils of the *Eucalyptus* genus. Additionally, *B. cinerea* was sensitive to lower or equal concentrations of cinnamon and clove extracts, compared to the antifungal concentrations of various essential oils described by Fontana et al. [5].

Considering that *B. cinerea* demonstrated morphological differences while grown on a different medium [24], changes of morphological attributes could be expected for isolates grown on a medium with an additional factor—plant extract. Although the *B. cinerea* isolates complex was highly sensitive to cinnamon extract, only a few isolates showed changed mycelium—even fewer changed the colour of the colonies. On the contrary, most complex isolates reacted to laurel extract treatment by changing the mycelium structure and colour despite the low sensitivity. We can assume that the possible antifungal mechanism of laurel extract was active as morphological attributes were affected, however, not strong enough to inhibit the growth of the pathogen. A changed surface and other features of *B. cinerea* hyphae were detected before [6,19]. In other investigations, structural alterations of *B. cinerea* hyphae were detected after exposure with *Eucalyptus* essential oils, causing the development of wrinkles, torsion, peeling and dehydration, and authors of the study suggested that hyphae changes are caused regarding essential oils action on the cell wall of the pathogen [11]. Our primary visual assessment of *B. cinerea* hyphae after treatment with extracts agree with other studies, as several changes were observed under microscope.

The conidia size in our control colonies of *B. cinerea* isolates varied and were smaller than described of the isolates in Kumari et al.’s [26] study. However, this feature may depend on each isolate’s individuality and the environmental conditions, including geographical region. Examples of how treatment with essential oils results in degraded hyphae with changed or damaged cell structures could be found in the literature [1]. Shrunk conidia, wrinkled, and deformed hyphae were observed on *B. cinerea* after *V. amygdalina* dichloromethane extract treatment [18]. Overall, cinnamon and clove extracts did not reduce the conidia size of the isolates complex in our study, except in the clove extract and isolate from the onion case. However, it was observed that there was a tendency after laurel extract treatment that half of the isolates germinated significantly smaller conidia, mostly at 2600 µL/L concentration. Yusoff et al. [18] suggest that the shrinkage of conidia could prevent the dispersion of the grey mould disease. To confirm the opposing results of the laurel extract impact on the morphology of *B. cinerea*, deeper morphological studies should be continued. In contrast to our study, no conidiation was observed in oil-treated samples of tomato *B. cinerea* [1].

To determine materials that may supplement chemical fungicides in the future, the effect on a wide range of plant host pathogens is essential. That is the reason why in this work we evaluated the sensitivity of the fungal pathogen *B. cinerea* complex, consisting of ten strains from different plant hosts to plant extracts, aiming to determine the possible plant-based materials with a significant impact on the control of horticultural and ornamental pathogens.

## 4. Materials and Methods

### 4.1. Botrytis Cinerea Isolates Complex

As *Botrytis cinerea* is a harmful pest of horticultural and ornamental crops, isolates from various plant hosts were selected as an investigation object. Ten single-spore *B. cinerea* isolates (Table 4) belonging to the cryptic species group I (resistant to fenhexamid) and group II (sensitive to fenhexamid) were selected for the study with the purpose to investigate their sensitivity to potential biopesticides—plant extracts.

Single-spore isolates were obtained from infected parts of plants with visual symptoms of grey mould. Species and phylogenetic identification were performed under conditions specified in the study by Rasiukevičiūtė et al. [27]. The transposable element type of each isolate is presented in Table 1. Single spore isolates were stored in the Lithuanian Research Centre for Agriculture and Forestry, Institute of Horticulture, Plant protection laboratory pathogen collection, and renewed on the PDA two weeks before the start of the experiments.

### 4.2. Plant Extracts

Dried cinnamon bark (*Cinnamomum zeylanicum*), clove buds (*Syzygium aromaticum*) and laurel leaves (*Laurus nobilis*) were extracted using the CO_2_ extraction method as described in the previous study [28]. Composition of the volatile compounds responsible for antifungal activities of each extract was determined previously using gas chromatography/mass spectrometry [29]. The main component of the cinnamon extract was trans-cinnamaldehyde, clove—eugenol and eugenol acetate—, and laurel—eucalyptol and alpha-terpinyl acetate [29,30].

### 4.3. Determination of the Sensitivity to the Extracts

The sensitivity of *B. cinerea* isolates complex was tested for the following concentrations: cinnamon extract—100–800 µL/L—, clove extract—100–800 µL/L—, and laurel extract—2600–4200 µL/L. The experiment was designed with four replicates per each treatment concentration. Sterile PDA medium was prepared as specified in the manufacturer’s instruction and the appropriate amounts of the extracts were separately poured into the medium and mixed on the rotary shaker for 10 min. Prepared extract-enriched medium was poured into Petri dishes. Previously cut 7 mm diameter mycelium plug of *B. cinerea* (each isolate separately) was put in the centre of the Petri dish. No extracts were added to the control plates of the isolates. Plates with mycelium plugs were placed for incubation at 22 °C in the dark for seven days. After seven days, radial colony growth (cm) of the *B. cinerea* isolates was measured. If radial colony growth was significantly reduced compared to control treatment, isolate expressed sensitivity to the tested concentration of the extract. If radial colony growth was fully reduced (equal to zero), isolate was considered as highly sensitive to the treatment.

### 4.4. Examination of Morphological Attributes of the Isolates Complex

Only concentrations with visual pathogen growth were included in the morphological attributes’ evaluation. Seven days after inoculation, fungal colonies treated with different extract concentrations visually compared to the ones in the control plates. Examined morphological attributes were mycelium and colour, evaluating the front and reverse sides of the colonies. Differences from the control plate were marked as described:

Mycelium:

(+)—extract treatment caused mycelium changes compared to the control plate.

(–)—fungal colony in the extract-treated plate matches the type of mycelium with the control plate.

Colour:

(+)—extract treatment caused different pigmentation compared to the control plate.

(–)—fungal colony in the extract-treated plate matches the colour with the control plate.

Furthermore, all colonies with visual fungal growth were examined for the presence of conidia. An amount of 1 mm of fungal mycelium was taken from the margin of the colony with a sterile needle and placed in 50% lactophenol blue solution on a microscope glass slide, mixed and covered with a glass coverslip and examined at 40× magnification using a phase-contrast light microscope Nikon Eclipse 80i (Nikon Instrument Inc., Melville, NY, USA). In case conidia were formed, the length and width (µm) of 30 conidia were measured using NIS-Elements D 3.2 programme (Nikon Instrument Inc., Melville, NY, USA). Four measurements per treatment were performed. The same program was used to capture observed morphological changes of hyphae.

### 4.5. Statistical Analysis

Statistical analysis was performed with the SAS Enterprise Guide programme (SAS Institute Inc., Cary, NC, USA)). Differences between means were analysed using Duncan Multiple Range Test (*p* < 0.05). Principal component analysis was also performed to group the analysed isolates of *Botrytis cinerea* complex according to the sensitivity to each of the extracts.

## 5. Conclusions

In conclusion, the study revealed varied sensitivity of the complex of different plant hosts *B. cinerea* isolates from cryptic species group I and II to clove, cinnamon, and laurel extracts. Isolates had the highest sensitivity to cinnamon extract as their growth was completely reduced at the lowest concentrations from all tested extracts. Different plant hosts *B. cinerea* isolates were grouped statistically according to their sensitivity to plant extracts. Morphological attribute changes (colour, mycelium, and conidia size) after treatment with extracts were described. Contrasting results were obtained in laurel extract, as this extract had the most impact on the changes of morphological attributes. However, the isolates complex was not susceptible to the investigated concentrations. This information gives possible directions for further studies evaluating the antifungal mechanism of oils. It is emphasized that the advantage of natural products from plants is their multicomponent composition providing a lower possibility for the pathogen to develop resistance quickly. Based on the results in our study, the cinnamon extract has promising antifungal properties, causing sensitivity to *B. cinerea*, infecting various plant hosts.

## Figures and Tables

**Table 1:** Comparison of the sensitivity of *Botrytis cinerea* isolate complex (BC1-BC10) to different concentrations of the extracts (µL/L) 7 days after inoculation. Data presented as radial colony growth of pathogens (cm).

Extract	µL/L	BC1	BC2	BC3	BC4	BC5	BC6	BC7	BC8	BC9	BC10
Cinnamon	100	7.80a	7.80a	7.80a	7.80a	7.80a	7.80a	7.80a	7.80a	7.80a	7.80a
	200	7.80a	7.80a	7.71a	7.80a	7.80a	7.80a	7.80a	7.80a	7.80a	7.54ba
	300	0.58g	7.80a	7.80a	7.80a	7.80a	dgfe	7.80a	7.80a	7.80a	7.45ba
	400	0.75g	7.80a	7.73a	6.61ba	7.76a	6.63ba	7.80a	7.80a	7.62a	6.55b
	500	0.00g	4.80dc	5.19c	0.86g	0.80i	0.00i	2.26f	0.70i	1.06fe	1.76jhi
	600	0.00g	0.00g	0.00e	0.00g	0.00i	0.00i	0.00g	0.00i	0.00f	0.00k
	800	0.00g	0.00g	0.00e	0.00g	0.00i	0.00i	0.00g	0.00i	0.00f	0.00k
Clove	100	7.80a	7.80a	7.80a	7.08ba	7.80a	7.80a	7.80a	7.80a	7.80a	6.69b
	200	7.14a	6.30b	6.96ba	6.91ba	6.01b	7.08ba	7.13a	6.91b	5.48cb	4.41dc
	300	5.29b	4.56dce	5.35c	6.90ba	4.79ed	3.50dfe	5.61cb	6.19cb	4.29cd	3.56dfe
	400	5.25b	4.08dce	3.05d	4.38dce	3.68fg	5.25bc	3.70e	4.35fg	3.63d	3.87dce
	500	0.00g	2.58f	0.00e	2.83f	3.28g	0.00i	4.55ced	3.71g	2.14e	2.45ghf
	600	0.00g	0.02g	1.22e	1.29g	2.24h	1.61hgi	1.44f	2.01h	1.77e	1.38ji
	800	0.00g	0.00g	0.00e	0.00g	0.00i	0.99hi	0.00g	0.00i	0.13f	0.91jk
Laurel	2600	4.68cbd	4.56dce	6.93ba	6.63ba	5.30cbd	6.65ba	4.75cbd	5.93cd	5.61b	4.78c
	2800	2.91efd	4.38dce	6.42bac	4.95dc	5.80cb	6.18ba	5.08cbd	5.77cde	5.27cb	4.43dc
	3000	3.08cefd	3.90dfce	5.30c	4.48dce	4.74ed	5.54bc	4.61ced	4.98fe	4.30cd	3.84dce
	3200	4.89cb	3.29dfe	6.58bac	3.49fe	5.10cd	2.81dgfe	4.66ced	4.93fe	4.79cbd	2.95gfe
	3400	1.93gef	4.80dc	5.25c	4.86dce	5.51cbd	5.59bc	5.29cb	5.08fde	5.65b	2.99gfe
	3600	1.44gf	3.08fe	5.98bc	4.75dce	4.24fe	2.11hgfe	5.77b	4.45fg	4.81cbd	2.24ghi
	3800	1.46gf	3.25fe	5.19c	4.80dce	4.06feg	3.86dce	5.01cbd	5.07fde	4.85cbd	3.11gfe
	4000	0.79g	4.08dce	5.10c	3.99dfe	3.83fg	1.84hgf	4.14ed	4.59f	4.51cbd	2.73ghfe
	4200	3.41cebd	4.84c	3.51d	5.74bc	5.45cbd	4.35dc	5.58cb	5.76cde	4.89cbd	3.86dce

Different letters show statistical differences of each isolate sensitivity to extract treatment according to the Duncan Multiple range test at the 5% probability level (*p* < 0.05).

**Table 2:** Mycelium and colour changes in *B. cinerea* isolate complex after treatment with cinnamon, clove, and laurel extracts. Colony morphology illustrations represent a surface side of *B. cinerea* grown for 7 days on PDA with investigated extract, at 22 °C in the dark.

Isolate	Cinnamon Extract			Clove Extract			Laurel Extract		
	M	C	CM	M	C	CM	M	C	CM
BC1	−	−	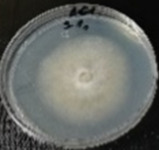	+	+	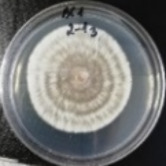	+	−	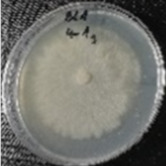
BC2	−	−	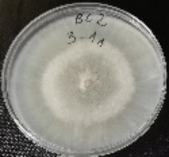	+	−	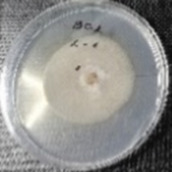	−	−	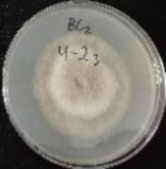
BC3	+	−	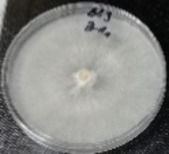	+	−	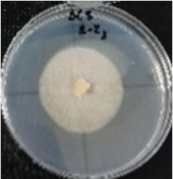	+	−	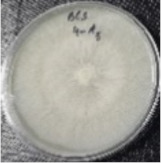
BC4	−	−	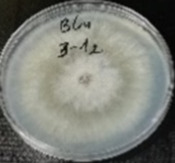	+	−	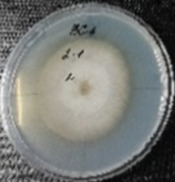	+	−	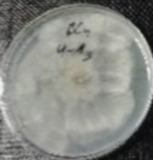
BC5	−	+	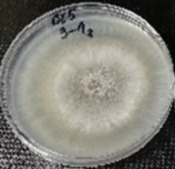	+	+	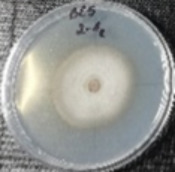	+	+	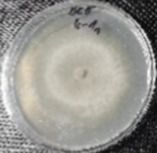
BC6	−	−	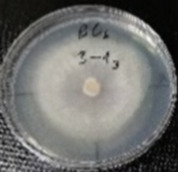	−	−	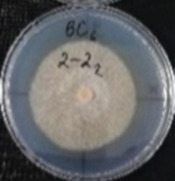	−	−	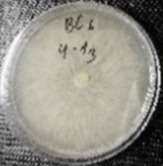
BC7	+	+	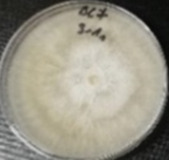	+	+	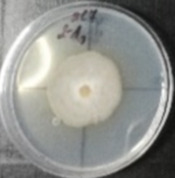	+	+	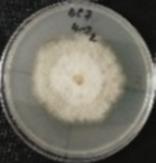
BC8	+	−	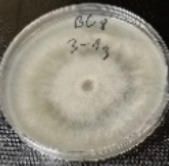	−	−	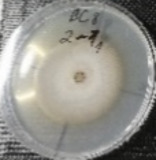	+	+	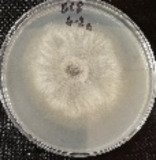
BC9	−	−	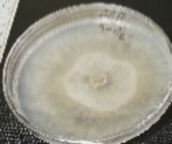	+	−	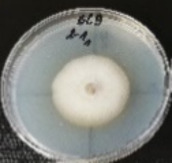	+	+	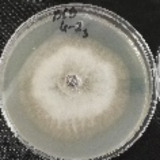
BC10	−	+	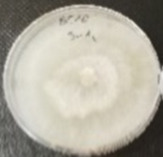	−	−	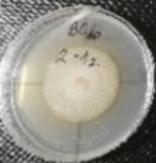	+	−	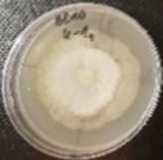

M—mycelium; C—colour; CM—colony morphology. “+” Visible changes of the morphological attribute. “−“ No visible changes of the morphological attribute.

**Table 3:** Conidia size (µm) of *B. cinerea* isolates after treatment with cinnamon, clove, and laurel extracts.

Extract Isolate		Control	Cinnamon		Clove		Laurel		
		0 µL/L	400 µL/L	600 µL/L	400 µL/L	600 µL/L	2600 µL/L	2800 µL/L	3000 µL/L
BC2	Length	19.38a	18.93a	–	13.56b	–	14.82b	n.d.	19.60a
	Width	14.54ba	16.05a		11.87b		12.56b		15.72a
BC3	Length	n.d.	19.91a		n.d.		19.07a		n.d.
	Width		16.05a				12.34b		
BC4	Length	19.92dc	22.13a		19.00ed	20.30bdc	21.02bac	17.66e	21.76ba
	Width	15.43b	13.03c		15.66b	16.39ba	15.45b	16.15ba	17.31a
BC5	Length	19.54ba	21.39a		19.75ba	18.24ba	20.31ba	16.94b	21.07a
	Width	15.24ba	15.87a		15.22ba	15.51ba	16.62a	13.15b	15.56ba
BC7	Length	20.91b	19.87b		n.d.	–	19.26b	24.70a	n.d.
	Width	14.60b	13.81b				16.46b	21.08a	
BC8	Length	19.13a	20.35a		20.53a	20.58a	20.92a	19.09a	16.75b
	Width	15.82ba	15.55ba		16.06a	15.30ba	16.62a	16.15a	14.08b
BC9	Length	18.43bac	17.85bc	20.35a	17.31c	17.83bc	19.92ba	17.52bc	18.09bac
	Width	14.34bc	14.59bc	15.09bac	13.82c	14.21bc	16.21a	13.77c	15.42ba
BC10	Length	17.36a	17.45a	–	n.d.	–	17.22a	17.74a	n.d.
	Width	13.55a	14.87a				13.78a	14.70a	

“n.d.” Means not detected. “–“ Sign means that no colony growth of the pathogen was observed. Different letters show statistical differences of each isolate conidia length/width after treatment with extracts according to the Duncan Multiple range test at the 5% probability level (*p* < 0.05).

**Table 4:** Origin, cryptic species groups, and genotypes of the *Botrytis cinerea* isolates complex used in the study.

Isolate Code	Plant Host	Latin Name	Group		Transposable Element Type			
			I	II	F	B	V	T
BC1	Strawberry	*Fragaria magna* f. *ananassa*	+				+	
BC2	Onion	*Allium cepa*		+		+		
BC3	Tomato	*Solanum lycopersicum*		+	+			
BC4	Raspberry	*Rubus idaeus*		+				+
BC5	Cabbage/leaf	*Brassica oleracea*		+				+
BC6	Apple	*Malus domestica*		+			+	
BC7	Grapevine	*Vitis* spp.		+				+
BC8	Bell pepper	*Capsicum annuum*		+				+
BC9	Leek	*Allium porrum*		+			+	
BC10	Rose	*Rosa* spp.		+			+	
Total			1	9	1	1	4	4

F—*Flipper*, B—*Boty,* V—*Vacuma*, T—*Transposa*, I—group—resistant to fenhexamid, II—sensitive. “+” indicates cryptic species group/transposable element type of the isolate.
